# Lip Verrucous Tumor in a Male With Chronic Graft-Versus-Host Disease

**DOI:** 10.7759/cureus.83953

**Published:** 2025-05-12

**Authors:** Hiroki Yamamoto, Teruyoshi Hisamoto, Jun Omatsu, Shinichi Sato

**Affiliations:** 1 Department of Dermatology, University of Tokyo Graduate School of Medicine, Tokyo, JPN

**Keywords:** chronic gvhd (cgvhd), cutaneous verrucous carcinoma, graft-versus-host disease (gvhd), hematopoietic stem cell transplantation (hsct), lower lip, squamous cell carcinoma (scc), verruciform xanthoma

## Abstract

Verruciform xanthoma (VX) is a rare benign tumor that usually occurs in the oral area or the urogenital region. Although VX sometimes arises in the oral space, VX on the lip is rare. The ratio of oral VX in chronic graft-versus-host disease (cGVHD) patients might be higher than that of the general population, and the frequency of lip cases may also be higher in cGVHD patients. In some cases, VX mimics malignant tumors such as verrucous carcinoma or squamous cell carcinoma. It is important to distinguish VX from malignant tumors to prevent excessive excision. Our case is a VX on the lower lip of a cGVHD patient. It was diagnosed by skin biopsy and treated by total excision. When we find a verrucous or papillary tumor, especially in a cGVHD patient, VX should be a candidate for diagnosis, as well as verruca vulgaris or verrucous carcinoma, to prevent unnecessary extensive excision.

## Introduction

Verruciform xanthoma (VX) is a rare benign tumor that usually occurs in the oral area or the urogenital region [1]. Its pathogenesis is unclear, but some underlying chronic inflammation is thought to induce VX [2]. It sometimes arises secondarily to lichen planus, lichen sclerosis, chronic graft-versus-host disease (cGVHD), discoid lupus erythematosus, and pemphigus vulgaris [2-3]. Past trauma or recurrent injury can also be a cause [3]. VX sometimes arises in the oral space, but VX on the lip is rare [4-5]. Especially, VX on a vermilion lip is even rarer.

Herein, we report VX on the lower vermilion lip of a cGVHD patient and discuss its rarity and its relationship to cGVHD.

## Case presentation

**Figure 1:**
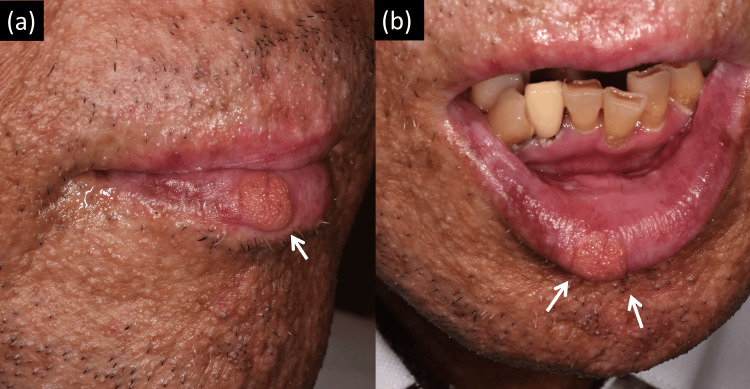
Clinical photographs (a) As indicated by the arrow, a granular tumor was observed on the lower vermilion lip. It was slightly elevated, and its borderline was clear, without crust, scale, or erosion. (b) No apparent change was seen on the mucosal side of the lip.

A 54-year-old male consulted us for a lower lip tumor that had gradually developed in the last few months. He had no habitual behaviors, such as frequently biting the lip, that could have caused trauma to the lip. He had been diagnosed with mycosis fungoides (MF) 10 years before and received several treatments, resulting in a poor response. He received allogeneic peripheral blood stem cell transplantation at the Department of Hematology and Oncology two years ago. After that, he had erythema on the trunk, arms, and legs. Blood test showed hepatic impairment. He was diagnosed with chronic graft-versus-host disease (cGVHD). Until now, he is being treated for chronic cGVHD and is receiving medication for cyclosporine, acyclovir, atovaquone, fluconazole, ursodeoxycholic acid, and vonoprazan. On the lower lip, a roughly round and slightly elevated tumor was observed (Fig. 1a). It was about 10 mm in diameter, and its surface was granular with no crust or scale. No erosion or ulcer was seen. It was asymptomatic, and the mucosal lip and oral mucosa had no apparent change (Fig. 1b).

**Figure 2:**
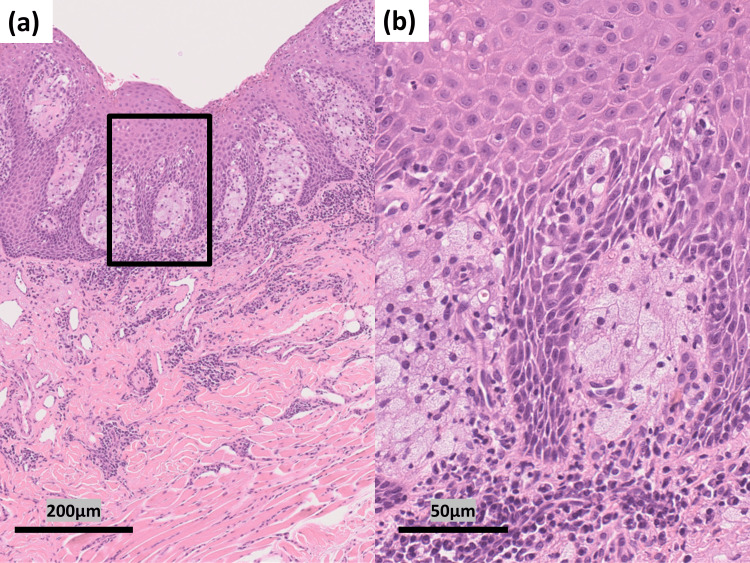
Histopathology of the skin sample obtained by punch biopsy Histopathology of the skin sample obtained by punch biopsy (hematoxylin–eosin staining, original magnifications: (a) ×100, (b) ×400). The box in the panel (a) is the high-magnification area shown in the panel (b): (a) Hyperkeratosis and elongation of rete edges are seen. Basophilic cell infiltration with dilation of capillaries is observed in the dermis. Bar = 200 μm. (b) Foamy xanthoma cells filled the papillary dermis, and plasma cells infiltrated the reticular layer of corium. Bar = 50 μm.

We performed a punch biopsy. Histopathological findings showed hyperkeratosis, elongation of the rete ridges without atypia, and foamy histiocytes filling papillary dermis (Fig. 2a, 2b).

Based on the diagnosis of VX, total resection was performed. Its histopathological findings also affirmed VX. Until now, no recurrence has been seen for 20 months.

## Discussion

The surface of VX is keratinized, and it usually appears granular, verrucous, or papillary with white, yellow, or sometimes red surface [6]. In some cases, VX mimics other neoplasms such as verrucous vulgaris, condyloma acuminatum, and verrucous carcinoma. It is necessary to perform a biopsy to distinguish VX from other tumors. In the histopathological examination, hyperkeratosis, focal parakeratosis, and verrucous acanthosis without epithelial atypia are seen; the most significant and diagnostic finding is the presence of xanthoma cells (foam cells) in the dermis [2,7]. These xanthoma cells contain plenty of lipids, and CD68, CD163, and CD63 are positive in immunohistochemistry, indicating a macrophage phenotype. VX sometimes arises in the oral space, but VX on a lip is rare [4-5]. Especially, VX on a vermilion lip is even rarer. Belknap et al. reported that among the total 212 cases of oral VX cases, only four were located on a lip, in contrast to 110 cases of gingiva as the most common location [4]. It is also reported that six cases of VX on the lower lip were previously published in the English literature [8]. On the other hand, VX secondary to cGVHD is also analyzed. Ori Bar et al. reviewed oral VX in cGVHD patients and described 12 cases [9]. They contained two lip VXs, a case of a 22-year-old male and of an 11-year-old male, and this ratio of two out of 12 cases is higher than the ratio of four out of 212 cases reported by Belknap et al. [4,10-11]. 

Basal keratinocytes of the epithelium in cGVHD patients are frequently seen with lichenoid destruction microscopically, which is thought to be of pathogenesis of VX [10]. As a result, the frequency of lip cases may be higher in cGVHD patients even if there is no injury in their lips, although it is only a hypothesis. Squamous cell carcinoma (SCC) also occurs in cGVHD patients more frequently than in post-hematopoietic stem cell transplantation patients without cGVHD [12]. When we find a verrucous or papillary tumor, especially in a cGVHD patient, VX should be a candidate for diagnosis, as well as verruca vulgaris or verrucous carcinoma. The common treatment for VX is simple excision, which has a good prognosis [1]. Cryotherapy, carbon dioxide laser, or radiation are also performed, but the treatment algorithm among them is not yet determined [1]. On the other hand, SCC should be excised with surgical margins [13]. Therefore, an accurate diagnosis will affect the postoperative cosmetic aspect.

## Conclusions

This case underscores the rare presentation of VX on the vermilion lip, particularly in a patient with cGVHD. Accurate diagnosis via histopathology, identifying characteristic foamy histiocytes, is essential to differentiate VX from malignancies like squamous cell carcinoma, especially in cGVHD patients who may have a higher incidence of lip VX. Simple surgical excision remains the standard treatment with a favorable prognosis, emphasizing the need for correct diagnostic consideration of such lesions in this patient population.
